# Poor sleep quality and daytime sleepiness in medical students: role of late bedtimes

**DOI:** 10.1590/1806-9282.20250630

**Published:** 2025-12-05

**Authors:** Aldo da Silva Oliveira, Luís Alberto Maciel Porto, Mariana Sousa Ibiapina, Karla Ianara Silva Tavares, Lanni Sarmento da Rocha, Edoarda Vasco de Albuquerque Albuquerque, Luciane de Souza Medeiros, Adriana Ximenes-da-Silva

**Affiliations:** 1 Universidade Federal de Alagoas, Faculty of Medicine – Maceió (AL), Brazil.; 2 Universidade Estadual de Ciências da Saúde de Alagoas – Maceió (AL), Brazil.; 3 Universidade Federal de Alagoas, Instituto de Ciências Biológicas e da Saúde – Maceió (AL), Brazil.; 4 Hospital Universitário Professor Alberto Antunes – Maceió (AL), Brazil.

**Keywords:** Students, medical, Sleep wake disorders, Sleep deprivation, Circadian rhythm, Sleep hygiene, Questionnaires

## Abstract

**OBJECTIVE:**

This study assessed sleep quality and habits affecting sleep among medical students in Alagoas, Brazil.

**METHODS:**

An online survey was conducted using the Pittsburgh Sleep Quality Index and the Epworth Sleepiness Scale, and a questionnaire on caffeinated beverage and calming herbal tea intake, medication use, and daily screen time was administered.

**RESULTS:**

A total of 165 students participated, 56.36% of whom were female. The Pittsburgh Sleep Quality Index results showed that 58.2% were poor sleepers, 15.2% had poor sleep quality, and 26.7% were good sleepers. Only 36% met the recommended 7–9 h of sleep per day. Shorter sleep duration correlated with poorer sleep quality and increased daytime sleepiness (rho=-0.5). No significant sex differences were observed. Participants were categorized as early-to-bed (51.5%, 23.9±4.9 years; 62% women) and late-to-bed (48.5%, 23.7±5.7 years; 50% women). The late-to-bed group showed poorer sleep quality, greater daytime sleepiness, and 1.4 h less sleep than the early-to-bed group (p<0.01). A later sleep schedule was significantly associated with poorer sleep quality, shorter duration, and greater daytime sleepiness (p<0.01). The semester of study did not significantly influence bedtime (p=0.45). No significant group differences were found in beverage or medication use.

**CONCLUSION:**

This study highlights the need for targeted interventions to improve sleep quality and duration by addressing late bedtimes and contributing factors to daytime sleepiness.

## INTRODUCTION

Sleep is a vital physiological process essential for neurocognitive functioning, emotional regulation, cellular repair, and the maintenance of overall health and quality of life^1^. Among undergraduate students in the health sciences, however, adequate sleep hygiene has become increasingly challenging. Frequent reports of reduced sleep duration, persistent fatigue, excessive daytime sleepiness, and impaired concentration indicate a pattern of sleep disruption that may compromise academic performance and psychological well-being^2^.

These disruptions are frequently linked to a combination of academic and extracurricular demands, including study obligations and irregular schedules associated with clinical training. The cumulative impact of these stressors often leads to altered circadian rhythms and reduced sleep duration, as students attempt to fulfill both academic and professional responsibilities. Sleep quality appears particularly vulnerable among medical students. A recent meta-analysis involving over 59,000 students from 31 countries, including Brazil, showed that medical students experience significantly more severe sleep disturbances than their peers in other health-related fields^3^.

Notably, these sleep-related challenges often persist beyond undergraduate study, continuing through the demanding years of medical internship and into the professional phase of clinical practice^4^. Medical education in Brazil, particularly for students preparing for the public health sector, is marked by extended academic hours, intensive clinical exposure, and elevated psychological stress^2,5^. The 6-year curriculum generally spans basic and clinical sciences, followed by demanding internships, often requiring 30–50 h of weekly engagement.

Despite institutional measures such as revised curricular structures and designated rest areas, students frequently report pathological levels of somnolence. Studies worldwide have confirmed that poor sleep quality adversely affects both academic and clinical performance in this population^6^. Furthermore, the use of stimulants to prolong wakefulness and anxiolytics to manage stress has been documented, both of which may further disrupt circadian homeostasis^7^.

Chronically poor sleep hygiene, such as delayed bedtimes, has a compounding effect, with each hour of bedtime delay associated with a reduction of 14–33 min of total sleep^8^. Therefore, promoting consistent sleep routines and early intervention strategies is critical, particularly in populations with highly variable schedules.

Given the established role of sleep in academic success and mental health, this study aimed to assess sleep quality and identify behaviors that disrupt sleep among medical students in public universities across the state of Alagoas, Brazil.

## METHODS

A cross-sectional study was conducted at the Universidade Federal de Alagoas (UFAL), a public university, between June and July 2023. Data collection used an online questionnaire divided into three sections: (1) sociodemographic information, electronic device use, beverage consumption, and extracurricular activities; (2) the Pittsburgh Sleep Quality Index-Portuguese version (PSQI-PT)^9^; and (3) the Epworth Sleepiness Scale (ESS)^10^. Students enrolled in medical programs at public institutions in Alagoas, Brazil, were eligible. Exclusion criteria included being aged under 18 years, having medical conditions known to impact sleep quality, or failure to consent or complete the questionnaire. The Ethics Committee of UFAL approved the study (CAAE: 66790822.5.0000.5013).

Google Forms hosted the questionnaire and was shared via email to coordinators, who then forwarded it to eligible medical students with a digital informed consent form (ICF). The ICF outlined study aims, risks, and voluntary participation.

The PSQI is a self-administered tool assessing sleep quality and disturbances over a 1-month period. A PSQI global score above 5 indicates poor sleep quality. The Portuguese version's seven-component scores demonstrated reliability (Cronbach's α=0.82). The ESS contains eight questions evaluating daytime sleepiness, with total scores from 0 to 24; scores of 11+ indicate excessive sleepiness.

The questionnaire included sociodemographic data and behavioral habits. It addressed extracurricular activities, caffeine intake, alcohol use to aid sleep, consumption of energy drinks or taurine-based supplements, use of calming herbal teas, and average daily screen time (<2 h, 2–4 h, 4–6 h, 6–8 h, and >8 h).

Sample size was calculated using G*Power software (version 3.1.9.7) for chi-squared goodness-of-fit tests (effect size ω=0.3; α=0.05; power=0.8), requiring 143 participants. Data were organized and analyzed using Excel, JASP (0.19.3), and JAMOVI (2.6.23).

Students were categorized as early-to-bed (before 11:00 PM) and late-to-bed (after 11:00 PM) groups. PSQI and ESS scores were summarized using means, standard deviations, and range. Descriptive statistics were used to analyze sleep duration and latency. Spearman's correlation and partial correlations assessed associations among PSQI, ESS, sleep duration, sex, and age.

Student's t-tests compared early vs. late sleepers for sleep metrics. Chi-squared or Mann-Whitney U test analyzed non-normal distributions and categorical variables. Fisher's exact test assessed academic semester effects. Gender and sex were separately controlled as confounders. P<0.05 and <0.01 were considered significant.

## RESULTS

A total of 165 medical students participated, with 93 (56.36%) identifying as female and 69 (43.64%) as male. Participants ranged in age from 18 to 49 years, with a mean age of 33.5 years. Majority (91%) were under 30 years old, averaging 22.5±2.8 years. A total of 11 students were aged between 30 and 36 years, and four were older than 40 years. Students were enrolled from the 1st to the 11th academic periods, with most (58.18%) in their first or second year, 33.33% in third or fourth, and 8.49% in fifth or sixth year.

**Figure 1:**
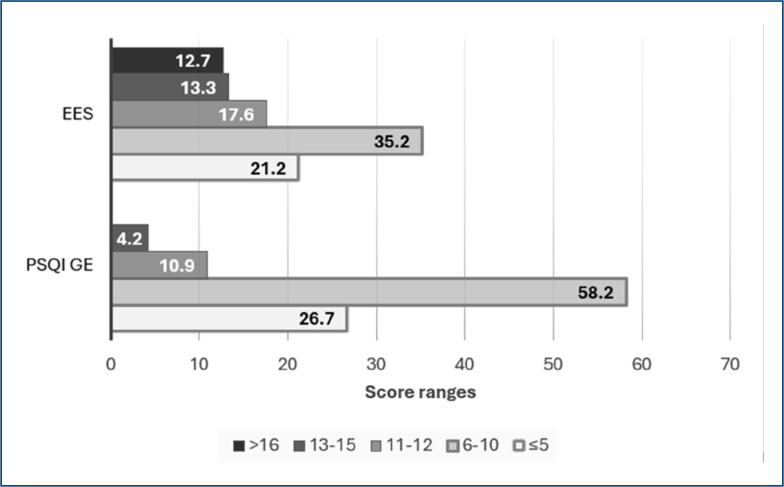
Percentage of medical students categorized according to combined Pittsburgh Sleep Quality Index and Epworth Sleepiness Scale score ranges. The score thresholds reflect gradations in sleep quality and daytime sleepiness, respectively. Ranges are as follows: 5 (good sleep quality/low sleepiness), 6–10 (fair sleep quality/mild sleepiness), 11–12 (moderate sleep quality/moderate sleepiness), 13–15 (poor sleep quality/high sleepiness), and >16 (very poor sleep quality/severe sleepiness). ESS: Epworth Sleepiness Scale; PSQI GE: Pittsburgh Sleep Quality Index Global Score.

The PSQI revealed that 26.7% of students were good sleepers (PSQI ≤5), 58.2% had poor sleep quality (scores 6–10), and 15.1% scored above 11, indicating significantly impaired sleep. ESS scores showed 21.2% with low daytime sleepiness (≤5), 35.2% with normal levels^6–10^, 17.6% with mild excessivesleepiness^11,12^, 13.3% with moderate excessive sleepiness^13–15^, and 12.7% with severe excessive sleepiness (>16) (Figure 1). These findings demonstrate a high prevalence of poor sleep quality and excessive daytime sleepiness.

The mean PSQI was 7.4±2.9 (range: 2.0–15.0) and mean ESS was 9.9 (range: 1.0–23.0), reflecting heterogeneous sleep patterns with many students reporting impaired sleep and notable sleepiness.

Students reported an average sleep duration of 6.5±1.2 h over the prior month, ranging from 2.5 to 10 h. Only 36% met the recommended 7–9 h per night. About 26.2% reported 5–6 h of sleep, another 26.2% reported 6–7 h, and 10.3% reported fewer than 5 h. Only 2.4% reported sleeping under 4 h, and 1.2% exceeded 9 h (Table 1).

**Table 1:** Results of the Epworth Sleepiness Scale score, Pittsburgh Sleep Quality Index global score, self-reported sleep duration, and sleep latency for the two groups (early-to-bed: asleep by 11 p.m.; late-to-bed: asleep after 11 p.m.)—Student's t-test.

	Group	n	Mean (±SD)	Mean difference	95%CI	Cohen's d	p
Epworth score	Early-to-bed	85	9.0 (±4.4)	−1.57	−3.36 to −0.49	−0.4	<0.001
	Late-to-bed	80	10.9 (±4.9)				
PSQI global score	Early-to-bed	85	6.6 (±2.8)	−1.92	−2.42 to −0.72	−0.6	<0.001
	Late-to-bed	80	8.2 (±2.7)				
Sleep latency	Early-to-bed	84	24.2 (±19.4)	1.47	1.18 to 1.76	0	0.7
	Late-to-bed	80	25.4 (±24.2)				
Total sleep period	Early-to-bed	84	7.2 (±0.9)	−1.22	−7.98 to 5.53	1.6	<0.001
	Late-to-bed	80	5.8 (±1.0)				

D: standard deviation; CI: confidence interval; PSQI: Pittsburgh Sleep Quality Index.

Spearman's correlation revealed a moderate negative relationship between sleep duration and PSQI score (rho=-0.5, p<0.01), indicating that shorter sleep duration was linked to poorer sleep quality. A weak negative correlation also emerged between sleep duration and ESS score (rho=-0.2, p<0.01), suggesting that less sleep was associated with greater sleepiness. A positive correlation was found between PSQI and ESS scores (rho=0.3, p<0.01), and ESS scores correlated negatively with age (rho=-0.2, p<0.05). No significant sex differences were observed.

Among respondents, 51.5% were categorized into early-to-bed (23.9±4.9 years; 62% women) and 48.5% as late-to-bed (23.7±5.7 years; 50% women) groups. Late sleepers reported poorer sleep quality, greater sleepiness, and averaged 1.4 h less sleep (p<0.01) (Table 1).

Comparisons across PSQI components (C1–C7) showed significant differences between early and late sleepers for C1, C3, and C7 (p<0.01), indicating poorer sleep quality, shorter sleep duration, and greater sleepiness in the late-to-bed group (Table 1).

No significant differences in beverage or medication use were found between groups (p>0.05). Similarly, energy drink intake showed a trend toward higher consumption in late sleepers (p=0.07). The academic semester did not influence bedtime (p=0.45) (Table 2).

**Table 2:** Comparison of screen exposure time, and consumption of coffee, energy drinks, relaxing teas, and hypnotics between the early-to-bed and late-to-bed groups.

Group		Time screen						
		**<2 h**	**2–4 h**	**4–6 h**		**6–8 h**	**>8 h**	**Total**
Early-to-bed	n	2	9	27		26	21	85
	% of total	1.2	5.5	16.4		15.8	12.7	51.5
Late-to-bed	n	0	7	17		27	29	80
	% of total	0	4.2	10.3		16.4	17.6	48.5
**Fisher's exact test, p=0.24**								
**Group**		**Coffee consumption**						
		**No**	**Yes**		**Total**			
Early-to-bed	n	36	49		85			
	% of total	21.8	29.7		51.5			
Late-to-bed	n	25	55		80			
	% of total	15.2	33.3		48.5			
**Chi-squared test, p=0.14**								
**Group**		**Consumption of energy drinks**						
		**No**	**Yes**		**Total**			
Early-to-bed	n	65	20		85			
	% of total	39.4	12.1		51.5			
Late-to-bed	n	51	29		80			
	% of total	30.9	17.6		48.5			
**Chi-squared test, p=0.07**								
**Group**		**Consumption of relaxing teas**						
		**No**	**Yes**		**Total**			
Early-to-bed	n	63	22		85			
	% of total	38.2	13.3		51.5			
Late-to-bed	n	62	18		80			
	% of total	37.6	10.9		48.5			
**Chi-squared test, p=0.61**								
**Group**		**Consumption of hypnotics**						
		**No**	**Yes**		**Total**			
Early-to-bed	n	78	7		85			
	% of total	47.3	4.2		51.5			
Late-to-bed	n	69	11		80			
	% of total	41.8	6.7		48.5			
**Chi-squared test, p=0.25**								

## DISCUSSION

Sleep deprivation among medical students is a well-recognized global concern. Evidence consistently links inadequate sleep with academic underperformance, emotional dysregulation, and long-term health consequences. Similar studies from countries such as Saudi Arabia report comparable rates of poor sleep quality and mental health challenges in this population^11^. In our study of public university medical students in Alagoas, Brazil, only 36% met the recommended 7–9 h of sleep, aligning with international sleep guidelines^12^. Many students reported chronic sleep insufficiency, which may compromise cognitive function and psychological well-being.

The impact of reduced sleep is considerable. Even modest sleep restriction impairs performance and safety, including risks to work productivity and driving^13^. Given sleep's role in memory, emotional stability, and learning, this study sought to assess sleep quality and identify behaviors contributing to disruption. Our findings echo previous reviews that have shown strong associations between poor sleep hygiene, psychological distress, and academic difficulties among university students^14,15^.

In our sample, later bedtimes correlated with shorter sleep duration and poorer sleep quality. Students who went to bed after 11 p.m. experienced significantly more sleep disruptions and daytime sleepiness. Interestingly, the academic semester was not statistically associated with bedtime patterns. However, variables such as employment, marital status, and commute distance were not evaluated, which could also influence sleep habits.

The results support broader research showing university students often adopt delayed sleep schedules, which are linked to reduced sleep quality^16^. Early detection of poor sleep hygiene using validated tools such as the PSQI and ESS is vital, particularly during formative academic years. Behavioral recommendations — such as substituting screen use with paper reading — may help improve sleep latency and melatonin regulation^17^.

Our findings that students with later bedtimes slept approximately 1.4 h less and reported more daytime fatigue point to opportunities for behavioral intervention. Younger students also experienced higher levels of daytime sleepiness, possibly due to challenges adapting to demanding academic environments. This trend persisted across semesters and mirrors data from other Brazilian universities showing widespread sleep disturbances^18^.

Late sleepers demonstrated a tendency toward increased consumption of energy drinks, potentially as a response to fatigue. Although no significant group differences were found in caffeine intake, hypnotic use, or screen exposure, the late-to-bed group showed a marginally higher intake of stimulants. Environmental factors such as daylight exposure and genetic influences (e.g., PER3 gene regulation) may affect chronotype, yet in our sample, bedtime patterns did not appear impacted by such external cues—suggesting digital and academic routines take precedence^19–21^.

The weak correlation observed between sleep duration and ESS suggests that these tools measure different dimensions. Many individuals with chronic insomnia report poor sleep quality without corresponding sleepiness, likely due to hyperarousal. In contrast, disorders such as sleep apnea increase sleep pressure and elevate ESS scores. The PSQI–ESS relationship varies depending on population characteristics.

Given the low incidence of clinical sleep disorders in our young adult sample, behavioral and psychosocial factors appear to play a greater role in shaping sleep perception than physiological impairment. These findings underscore the urgency of implementing educational and institutional strategies to promote healthy sleep practices. Interventions tailored to medical students—such as digital use guidance, curriculum-based awareness programs, and supportive policies—may foster improved sleep hygiene, enhance resilience, and reduce daytime fatigue.

### Limitations

This study has some limitations. Self-reported questionnaires may be subject to memory and response biases, particularly when reporting sleep behaviors and stimulant use. The cross-sectional design limits causal inference. Underrepresentation of final-year students may reduce the representativeness of the sample. While stimulant use was more frequent among late sleepers, its impact on sleep quality could not be definitively determined.

## Data Availability

The datasets generated and/or analyzed during the current study are available from the corresponding author upon reasonable request.

## References

[1] Schneider L (2020). Neurobiology and neuroprotective benefits of sleep. Continuum (Minneap Minn).

[2] Rao WW, Li W, Qi H, Hong L, Chen C, Li CY (2020). Sleep quality in medical students: a comprehensive meta-analysis of observational studies. Sleep Breath.

[3] Carvalho VO, Conceição LSR, Gois MB (2020). COVID-19 pandemic: beyond medical education in Brazil. J Card Surg.

[4] Solis AC, Lotufo-Neto F (2019). Predictors of quality of life in Brazilian medical students: a systematic review and meta-analysis. Braz J Psychiatry.

[5] Maddalena NCP, Lucchetti ALG, Moutinho ILD, Ezequiel ODS, Lucchetti G (2024). Mental health and quality of life across 6 years of medical training: a year-by-year analysis. Int J Soc Psychiatry.

[6] Falloon K, Bhoopatkar H, Moir F, Nakatsuji M, Wearn A (2022). Sleep well to perform well: the association between sleep quality and medical student performance in a high-stakes clinical assessment. Sleep Adv.

[7] Alasmari MM, Alkanani RS, Alshareef AS, Alsulmi SS, Althegfi RI, Bokhari TA (2022). Medical students’ attitudes toward sleeping pill usage: a cross-sectional study. Front Psychiatry.

[8] Grummon AH, Sokol RL, Lytle LA (2021). Is late bedtime an overlooked sleep behaviour? Investigating associations between sleep timing, sleep duration and eating behaviours in adolescence and adulthood. Public Health Nutr.

[9] Bertolazi AN, Fagondes SC, Hoff LS, Dartora EG, Miozzo IC, Barba ME (2011). Validation of the Brazilian Portuguese version of the Pittsburgh Sleep Quality Index. Sleep Med.

[10] Bertolazi AN, Fagondes SC, Hoff LS, Pedro VD, Menna Barreto SS, Johns MW (2009). Portuguese-language version of the Epworth Sleepiness Scale: validation for use in Brazil. J Bras Pneumol.

[11] Al-Khani AM, Sarhandi MI, Zaghloul MS, Ewid M, Saquib N (2019). A cross-sectional survey on sleep quality, mental health, and academic performance among medical students in Saudi Arabia. BMC Res Notes.

[12] Watson NF, Badr MS, Belenky G, Bliwise DL, Buxton OM, Consensus Conference Panel (2015). Joint consensus statement of the american academy of sleep medicine and sleep research society on the recommended amount of sleep for a healthy adult: methodology and discussion. Sleep.

[13] Dawson D, Sprajcer M, Thomas M (2021). How much sleep do you need? A comprehensive review of fatigue related impairment and the capacity to work or drive safely. Accid Anal Prev.

[14] Dutra Silva RC, Garcez A, Pattussi MP, Olinto MTA (2022). Prevalence and factors associated with excessive and severe daytime sleepiness among healthcare university students in the Brazilian Midwest. J Sleep Res.

[15] Ridner SL, Newton KS, Staten RR, Crawford TN, Hall LA (2016). Predictors of well-being among college students. J Am Coll Health.

[16] Pu Z, Ng ASC, Suh S, Chee MWL, Massar SAA (2025). Failing to plan: bedtime planning, bedtime procrastination, and objective sleep in university students. Sleep Med.

[17] Chang AM, Aeschbach D, Duffy JF, Czeisler CA (2015). Evening use of light-emitting eReaders negatively affects sleep, circadian timing, and next-morning alertness. Proc Natl Acad Sci U S A.

[18] Souza AKR, Sandes RS, Vasco RFV, Albuquerque EVA (2024). Quality of sleep and excessive daytime sleepiness among medical students in a Brazilian private university. Rev Assoc Med Bras (1992).

[19] Siudej K, Malinowska-Borowska J (2021). Relationship between chronotype and consumption of stimulants. Chronobiol Int.

[20] Leocadio-Miguel MA, Carneiro BT, Ximenes-da-Silva A, Caumo W, Grassi-Kassisse D, Pedrazzoli M (2018). PER3 gene regulation of sleep-wake behavior as a function of latitude. Sleep Health.

[21] Taebi M, Eghbali T, Balvardi M, Ghonchepour A, Azizzadeh Forouzi M, Hossienzadeh A (2025). Chronotype and sleep patterns among para-medical students at Kerman University of Medical Sciences: a cross-sectional study. Sleep Sci Pract.

